# Genome-wide identification and salt stress expression analysis of the PLATZ transcription factor genes in *Betula platyphylla*

**DOI:** 10.1270/jsbbs.24023

**Published:** 2024-11-20

**Authors:** Yang Li, Mingyu Yu, Yao Chi, Meiqi Zhou, Zihan Wang, Yan Gao, Xu Li, Caiqiu Gao, Chao Wang

**Affiliations:** 1 State Key Laboratory of Tree Genetics and Breeding (Northeast Forestry University), 26 Hexing Road, Harbin, 150040, China

**Keywords:** zinc finger protein, PLATZ transcription factor, *Betula platyphylla*, salt stress

## Abstract

The PLATZ (Plant AT rich protein and zinc binding protein) transcription factor, which is a type of plant specific zinc dependent DNA binding protein, participates in regulating the process of plant growth and environmental stress responses. In order to clarify the characteristics of the *PLATZ* family genes in birch (*Betula platyphylla*), the members of the *PLATZ* family were screened and analyzed in this study. Totals of ten *BpPLATZ* genes were identified in birch genome and classified into five groups base on phylogenetic relationship, *BpPLATZ* genes in the same group usually possess a similar motif composition, exon or intron number. These ten genes distributed on eight chromosomes of fourteen chromosomes of birch. In addition, various *cis*-elements were distributed in the promoter regions of *BpPLATZs*, especially with abundant MYC, ABRE and MYB, which were reported to be involved in salt stress responses. The RT-qPCR analysis results show that most genes have the higher expression levels in the roots compared to leaves and stems in birch. *BpPLATZ3*, *BpPLATZ5*, *BpPLATZ6*, *BpPLATZ7* and *BpPLATZ8* are significantly induced expressed response to salt stress. These studies provide a basis for the further functional study of the *BpPLATZ* genes.

## Introduction

Transcription factors (TFs) play a critical role in determining cell fate decisions by integrating developmental and environmental signals through binding to specific *cis*-regulatory modules and regulating spatio-temporal specificity of gene expression patterns (Hajheidari and Huang 2022). Zinc finger protein is one of the largest transcription factor families in plants. The PLATZ (Plant AT rich protein and zinc binding protein) transcription factor family is a type of plant specific zinc dependent DNA binding protein. The first member of the family, *PLATZ1*, was found in peas. These family members contain two separate zinc finger domains, namely C-x2-H-x11-C-x2-C-x(4-5)-C-x2-C-x(3-7)-H-x2-H and C-x2-C-x(10-11)-C-x3-C, which can bind non-specific to A/T rich sequences (Nagano *et al.* 2001). PLATZ have been found in a number of plants, including *Arabidopsis thaliana* (12), *Triticum aestivum* L. (62) (Fu *et al.* 2020), *Ginkgo biloba* L. (11) (Han *et al.* 2022), *Medicago sativa* L. (55) (Li *et al.* 2023), *Solanum lycopersicum* L. (24) (Zhang *et al.* 2023b), *Citrullus lanatus* L. (12) (Qi *et al.* 2023), and *Populus trichocarpa* (18) (Ma *et al.* 2023). However, there have been no reports on the characteristics and systematic analysis of the *PLATZ* family genes members in birch genome.

According to relevant research, the PLATZ transcription factor is involved in the process of plant growth and development. PLATZ transcription factor plays an important role in regulating plant cell proliferation and division, as well as growth and development processes (Kim *et al.* 2018). For example, The GmPLATZ may exert its functions through direct binding to the promoters and activation of the expression of cyclin genes and *GmGA20OX* for cell proliferation in *Glycine max* (Hu *et al.* 2023). GL6, encoding PLATZ in *Oryza sativa* positively regulates cell division to increase cell numbers of the spikelet hull, resulting in larger grains (Wang *et al.* 2019). PLATZ has essential roles in seed endosperm development, as well as promoting cell proliferation duration in the earlier stages of the crops (Fu *et al.* 2020). *PLATZ-A1* (*TraesCS6A02G156600*), which can modulate the effect of DELLA on wheat plant height, is expressed mainly in the elongating stem and developing spike (Zhang *et al.* 2023a). In addition, the PLATZ transcription factor is involved in abiotic stress and plays an important role in environmental stress responses. For example, PLATZ1 from *Gossypium hirsutum* responds to osmotic and salt stress during the germination and seedling stages of transgenic Arabidopsis (Zhang *et al.* 2018). Expression of *PtPLATZ3* significantly enhanced Cd tolerance and accumulation of transgenetic in *P. trichocarpa* (Ma *et al.* 2023). *AtPLATZ1* and *AtPLATZ2* can regulate seed dehydration tolerance, and PLATZ1 positively regulates drought resistance in nutrient tissues in wild-type Arabidopsis plants (González-Morales *et al.* 2016). Five *PLATZ* genes were highly expressed in multiple tissues of *Carya illinoensis* and strongly responded to drought, salt and heat stress (Zhang *et al.* 2023c). *SlPLATZ1* from *Solanum lycopersicum* was found to play an important role in salt stress (Zhang *et al.* 2023b). Although PLATZ has been shown to be involved in abiotic stress in some species, but the roles and functions of *PLATZ* genes in birch have not been elucidated.

*Betula platyphylla* is one of the most important pioneers and fast-growing tree species with important economic and ecological value in East Asia (Lyu *et al.* 2020). However, various environmental stresses have adverse effects on the growth and development of birch, including abiotic stresses such as salt, drought, and high temperature. In some areas where birch are mainly distributed, severe salinization has affected the afforestation and application. So improving the salt tolerance of birch is an urgent problem that needs to be solved. Transgenic methods can be used to improve plant stress resistance, therefore, screening genes related to salt tolerance is of great value. The genome sequence published in 2021 provides the possibility to identify and describe the whole *PLATZ* family in birch (Chen *et al.* 2021). In this study ten *PLATZ* genes were identified from birch genome. Their sequence characteristics were analyzed by bioinformatics methods. In addition, the expression levels of *PLATZ* family genes in roots, stems and leaves of birch, as well as under salt stress were analyzed using RT-qPCR analysis. These results laid the foundation for further understanding the structure and function of *BpPLATZ* genes, and provide candidate genes for further salt tolerant molecular breeding.

## Materials and Methods

### Identification and classification of *PLATZ* genes in birch

The sequences data was derived from the entire birch genome (Chen *et al.* 2021). The protein sequences containing the complete PLATZ domain of *B. platyphylla* were screened and retained using the Pfam database (http://pfam.xfam.org/) (Larkin *et al.* 2007). All PLATZ protein sequences and the presence of the PLATZ domain were identified using the NCBI CD-Search program (https://www.ncbi.nlm.nih.gov/Structure/cdd/wrpsb.cgi) and the InterPro online tool. The ProtParam tool in the online software ExPASy (Wilkins *et al.* 1999) (https://web.expasy.org/protparam/) was used to analyze the physicochemical properties of the selected members of the *PLATZ* family genes in birch. Sequences of *AtPLATZs* were download from the Tari (https://www.arabidopsis.org/), and sequences of *PtPLATZs* were download from the Phytozome (Goodstein *et al.* 2012) (https://phytozome-next.jgi.doe.gov/).

### Chromosomal distribution, duplication events and syntenic relationship analysis

Mapchart (Voorrips 2002) was used to determine the location of *BpPLATZ* family genes on chromosomes based on birch genomic information and visualized using TBtools (Chen *et al.* 2020). The genome information of two species, *A. thaliana* (GCF_000001735.4) and *P. trichocarpa* (GCF_000002775.4), were downloaded from NCBI (https://www.ncbi.nlm.nih.gov/). The collinearity relationship of *PLATZ* genes in *B. platyphylla* was visualizing using Circos. MCScanX (Wang *et al.* 2012). was used to analyze the collinear relationship of *PLATZ* genes among *B. platyphylla*, *A. thaliana* and *P. trichocarpa*.

### Phylogenetic analysis of the *BpPLATZ* family genes in birch

The PLATZ protein sequences encoded by 10 identified *PLATZ* genes of *B. platyphylla* were compared with 12 known PLATZ protein sequences of *A. thaliana* and 18 PLATZ protein sequences of *P. trichocarpa* using ClustalW (Krzywinski *et al.* 2009) program in MEGA-X (Kumar *et al.* 2018). The maximum likelihood (ML) method was used to construct three plant phylogenetic trees in MEGA-X software,with 1000 bootstrap replications. And use the online software Evolview (https://evolgenius.info//evolview-v2/#login) (He *et al.* 2016) to beautify the evolutionary tree.

### Protein structure analysis and subcellular localization prediction

The FASTA file of BpPLATZ proteins was submitted to SPOMA (https://npsa.lyon.inserm.fr/cgi-bin/npsa_automat.pl?page=/NPSA/npsa_sopma.html) for secondary structure prediction. 3D structure prediction of BpPLATZ proteins was provided by the Normal Model of Phyre2 Server (http://www.sbg.bio.ic.ac.uk/phyre2) (Kelley *et al.* 2015). Subcellular localization of BpPLATZ proteins was predicted using online software WoLF PSORT (https://wolfpsort.hgc.jp/) (Horton *et al.* 2007).

### Gene structure analysis, motif detection and *cis*-element predictions

The FASTA file of BpPLATZ proteins was submitted to the online software MEME (Bailey *et al.* 2009) (https://meme-suite.org/) to analyze the conserved Motif. The number of motifs was set to 10 and other parameters were default values. TBtools (Chen *et al.* 2020) was used to visualize the results. The PlantCARE (http://bioinformatics.psb.ugent.be/webtools/plantcare/html/) and GSDS 2.0 were used to analysis and extract the *cis*-element on promoter region of *BpPLATZ* genes.

### Plant materials and salt stress treatments

Birch plants (*B. platyphylla*) were grown in vitro on 1/2 MS medium at 25°C and approximately 60% humidity, with 16 hours of light and 8 hours of darkness per day. For tissue specific expression analysis, leaves, stems and roots of two month birch plantlets were collected respectively. For stress response analysis, plantles were divided into six groups, the treatment method adopts time reversal and the plantlets were transferred into a medium containing 0.2 M NaCl. The treatment for 48 h was processed first, then 24 h, 12 h, 6 h, 3 h. 48 h hours later, both the five groups of treated plants and the water treated plants (0 h, control) were collected at the same time and subjected to RT-qPCR. Each treatment contained three plantlets and was repeated three times. Then samples were stored at –80°C for subsequent use.

### RNA extraction and RT-qPCR analysis

Total RNA of the birch samples were extracted using the plant RNeasy Extraction Kit (BioTeKe, China), followed by electrophoresis on a 1% EB\ agarose gel to evaluate the quality of the total RNA. Primescript^TM^ RT kit (Toyobo, Osaka, Japan) was used to reverse transcribe the total RNA into cDNA, and dilute the 20 μl reaction system to 200 μl for RT-qPCR amplification. Use 2 × Real Star Green Power Mixture (Genstar) for RT-qPCR on MJ Research Optical Instrument (Bio-Rad, Hercules, CA, USA). The RT-qPCR reaction system was: green PCR Mix 10 μl, forward primer 1 μl, reverse primer 1 μl, diluted cDNA template 2 μl, deionized water 6 μl. The RT-qPCR reaction programs were: 94°C for 2 min; 28 cycles of 94°C for 30 s, 58°C for 30 s and 72°C for 45 s; and then 82°C for 1 s for plate reading. To ensure the reproducibility of the experimental results, RT-qPCR was carried out for three repeated experiments. The relative expression levels were calculated by the 2^–ΔΔCT^ method (Livak and Schmittgen 2001).

## Results

### Identification of *BpPLATZ* genes in birch

In order to identify *PLATZ* family genes in birch, the HMM file (PF04640) was used to search with the birch genome, and 11 *PLATZ* genes were screened out. NCBI CD-Search program and InterPro online tool were used to confirm the existence of conserved PLATZ domains and remove redundant sequences. Further identification was carried out using the reported *PLATZ* family genes in Arabidopsis and poplar, as well as selected *PLATZ* genes in birch. A total of ten sequences were identified as members of the PLATZ family in birch and named as *BpPLATZs* based on their successive chromosomal locations from top to bottom. The physicochemical properties analysis of the ten BpPLATZ proteins suggested that the number of amino acids of these proteins rang from 597 to 972 aa. The molecular weight of the proteins ranged from 49.04 kD to 81.04 kD and theoretical PI varied from 4.98 to 5.18 (Table 1). Among the ten proteins, the isoelectric PI of the protein sequence was less than 7, indicating that the ten BpPLATZ proteins belonged to acidic proteins. Total number of the proteins atoms ranged from 6304 to 10333. The instability index of these proteins were all greater than 30, indicating that the ten BpPLATZ proteins were all unstable proteins. Grand average of hydropathicity of all the proteins was positive, indicating that all the ten BpPLATZ proteins were hydrophobic proteins.

### Chromosomal distribution, duplication events and syntenic analysis of *BpPLATZ* genes

**Fig. 1.**
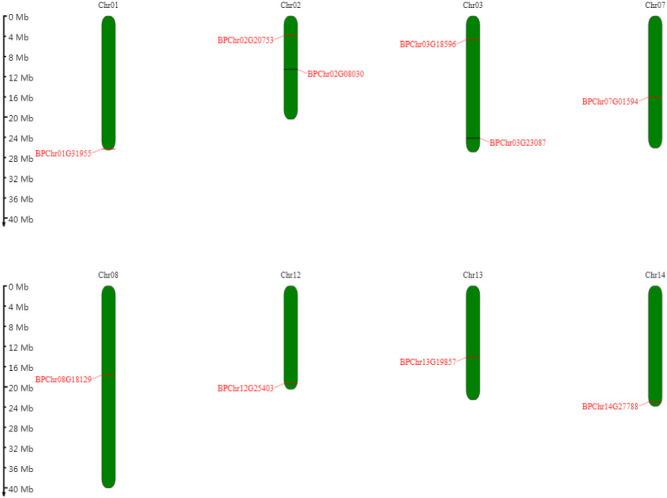
Mapping of the *BpPLATZ* genes on the chromosomes. The corresponding gene names are marked in red and written on both sides of each chromosome.

Mapchart software was used to determine the positions of *BpPLATZ* genes on chromosomes. The results showed that ten *BpPLATZ* genes were unevenly distributed on eight chromosomes out of fourteen chromosomes of birch (Fig. 1). Two genes each distributed on two chromosomes (Chromosome 02 (Chr02) and Chr03). Chr01, Chr07, Chr08, Chr12, Chr13, Chr14 contain one of the *BpPLATZ* family genes respectively.

**Fig. 2.**
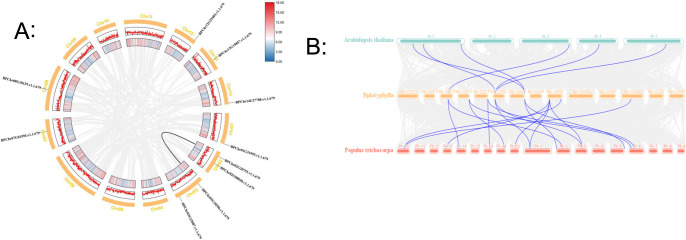
Collinearity analysis of the *BpPLATZ*. A: Collinearity analysis of *BpPLATZ* genes in birch. The circle plot represents the chromosomes of birch, and the identified collinear genes are linked by black line. B: Synteny analysis of *PLATZ* genes between birch and two other representative plant species. The blue lines represent the syntenic *PLATZ* gene pairs, while the gray lines in the background represent the collinear blocks between birch and two other representative species.

Gene duplication analysis can provide information regarding evolution during the expansion of *BpPLATZ* genes. In this study, the collinearity of *BpPLATZ* family genes was analyzed using the Circos tool in the TBtools software. Circos analysis showed that *BpPLATZ3* and *BpPLATZ4* were homologous and duplications distributed on two chromosomes (Fig. 2A). Through collinearity analysis of *PLATZ* family genes of *B. platyphylla*, *A. thaliana* and *P. trichocarpa* (Fig. 2B), it can be seen that there are six collinear pairs between *B. platyphylla* and *A. thaliana*, and sixteen collinear pairs between *B. platyphylla* and *P. trichocarpa*. *BpPLATZ1*, *BpPLATZ3* and *BpPLATZ9* were homologous with the other two species. The two genes (*BpPLATZ2* and *BpPLATZ6*) have no homology relationship with the other two species.

### Phylogenetic analysis of *PLATZ* genes

**Fig. 3.**
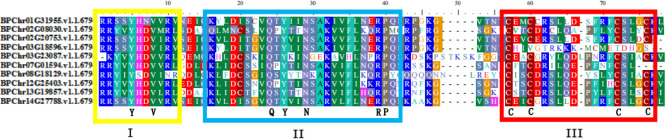
Amino acid sequence alignment analysis of BpPLATZ proteins. Note: Regions I, II, and III represent conserved regions of BpPLATZ proteins, the yellow area represents I, the blue area represents II, and the red area represents III.

Multiple alignment of the domain sequences of ten members of PLATZ family proteins of birch showed that the BpPLATZ proteins had three conserved regions (Fig. 3), namely regions I, II and III. In these ten proteins, eight amino acids belong to conserved amino acids, namely two Y (tyrosines), V (valine), Q (glutamine), N (aspartamide), R (arginine), P (proline) and C (cysteine). They remain unchanged in the *BpPLATZ* family genes, suggesting that these eight conserved amino acids play an important role in the coding of *BpPLATZ* genes. Meanwhile, the C-x2-C-x (10-11)-C-x3-C part of region III belongs to the zinc finger domain, which fully indicates that these proteins belong to plant specific zinc-dependent DNA binding protein.

**Fig. 4.**
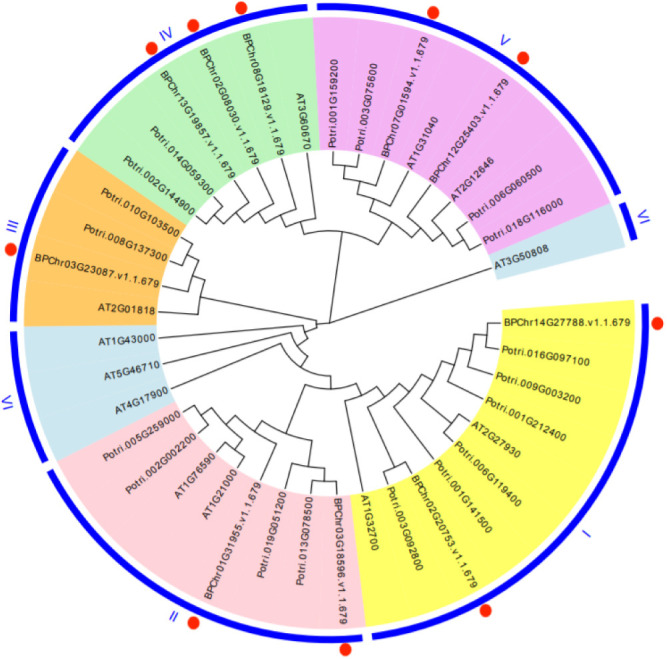
Phylogenetic relationship among *B. platyphylla*, *A. thaliana* and *P. trichocarpa*. 10 PLTZA proteins from birch, 12 PLATZ proteins from Arabidopsis and 18 PLATZ proteins from poplar were divided into 7 groups. The groups of birch were I–V, each group was assigned a background color. The solid red circle marks each birch gene.

MEGA5 software was applied to construct the phylogenetic tree based on the protein sequence encoded by *BpPLATZ* genes. Phylogenetic analysis of *PLATZ* genes from *B. platyphylla*, *A. thaliana* and *P. trichocarpa* showed that *BpPLATZ* genes could be clustered into five groups (I, II, III, IV, V) (Fig. 4), which were consistent with *PLATZ* gene groups of *P. trichocarpa* (Ma *et al.* 2023). *BpPLATZ3* and *BpPLATZ10* were in group I, *BpPLATZ1* and *BpPLATZ4* were in group II, *BpPLATZ6* and *BpPLATZ8* were in group V, only *BpPLATZ5* was in group III, as well as group IV included three genes *BpPLATZ2*, *BpPLATZ7* and *BpPLATZ9*. In group I, II, III and V, the ratio of protein number of birch and model plant *A. thaliana* was 1:1, but in group IV, the ratio of birch and *A. thaliana* was 3:1. Meanwhile, in group II, III, IV and V, the ratio of protein number between *B. platyphylla* and *P. trichocarpa* was 1:2, but in group I, the ratio of protein number between *B. platyphylla* and *P. trichocarpa* was 1:3.

### Protein structure analysis

The secondary structure prediction of the ten BpPLATZ proteins showed (Table 2) that all the ten proteins contained α-helix, β-corner, extended chain and random curl, the proportion of each part was significantly different. Among them, random coil accounted for the largest proportion, followed by α-helix and extension chain, β-angle accounted for the smallest proportion. Subcellular localization prediction by software WoLF PSORT results showed that all BpPLATZ proteins were predicted to locate in the nucleus, which indicates the characteristics as transcription factors of PLATZs. BpPLATZ1 was also predicted in cytoplasm, BpPLATZ2 was also predicted in chloroplasts and BpPLATZ7 was also located in mitochondria, suggesting that they perform different functions in different organelles.

**Fig. 5.**
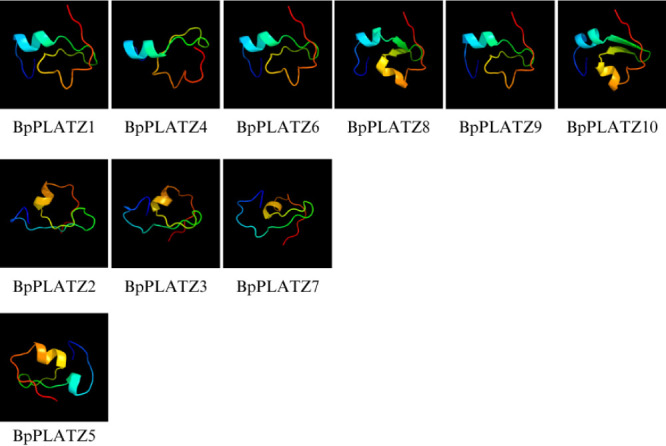
3D structure prediction of BpPLATZ proteins (the confidence is >90%). The first row of six protein structures represents d2dq5a1, the second row of three protein structures represents d2djaa1, and the third row of three protein structures represents d2csval.

3D structure predictions of BpPLATZ proteins generated by Phyre2 server were shown for all the ten BpPLATZs (Fig. 5) and all the proteins contained B-box zinc-binding domains. The prediction results indicate that the ten BpPLATZ proteins are divided into three types of protein templates (d2dq5a1, d2djaa1, d2csval), among which d2dq5a1 was the most common type, and the protein structure of BpPLATZ5, which in group III, is different from the other nine protein structures, which is d2csval template. This further confirms the accuracy of evolutionary tree grouping. The 3D structure of BpPLATZ1, BpPLATZ4, BpPLATZ6, BpPLATZ8, BpPLATZ9 and BpPLATZ10 were similar, which suggested that these 6 proteins might have similar functions.

### Conserved motifs, gene structure and *cis*-elements analyses of BpPLATZs

**Fig. 6.**
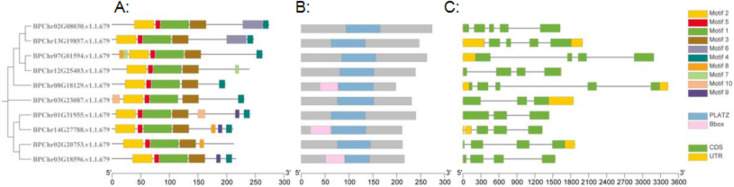
Phylogenetic analysis of PLATZ family genes and protein conserved motif analysis of *B. platyphylla*. A: Motif composition of PLATZ proteins. The 10 conserved motifs were shown in different color boxes, numbered Motif 1–10. B: Conserved domain composition of PLATZ proteins. C: The exon/intron structure of *BpPLATZs*.

The conserved motifs of BpPLATZ proteins were analyzed by online software MEME. The results show that, all the ten BpPLATZ domains contained motif 1, motif 2, motif 3 and motif 5 (Fig. 6A). Motif 4 is found in all members except BpPLATZ8 and BpPLATZ3. All genes in II group contain motif 9, while all genes in V group contain motif 7. The central region of all BpPLATZ contains PLATZ conserved domains (Fig. 6B) that provide zinc-dependent DNA binding capabilities. Among the ten *BpPLATZ* genes, different numbers of introns (3–5) were found (Fig. 6C). Within the same group, the difference does not exceed one. For example, in group IV, the number of introns is four or five, in group V the number of introns are all four. The exon number of all *BpPLATZ* genes is 0–2.

**Fig. 7.**
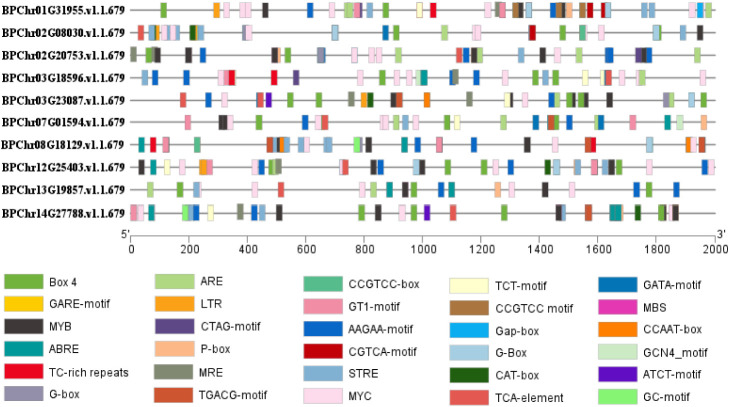
Analysis of *cis*-element of *PLATZ* gene family. This study cited 30 types of *cis*-elements, each represented by 30 different colors.

The *cis*-elements in the 2.0 kb promoter region upstream from the start codon of the *BpPLATZ* family genes were predicted (Fig. 7). The results show that in addition to common promoter *cis*-elements such as TATA-box (not shown) and CAAT-box (not shown), the upstream regulatory sequences of these genes also include defense and stress response elements such as MYC, MYB, LTR and GC-motif, light-responsive elements such as CTT-motif, GATA-motif, G-Box and others, plant hormone response elements such as CGTCA motifs, ABRE motifs, TCA-element and P-box, the maximum number is MYC *cis*-elements.

### Expression profiles of *BpPLATZ* genes across different tissues

**Fig. 8.**
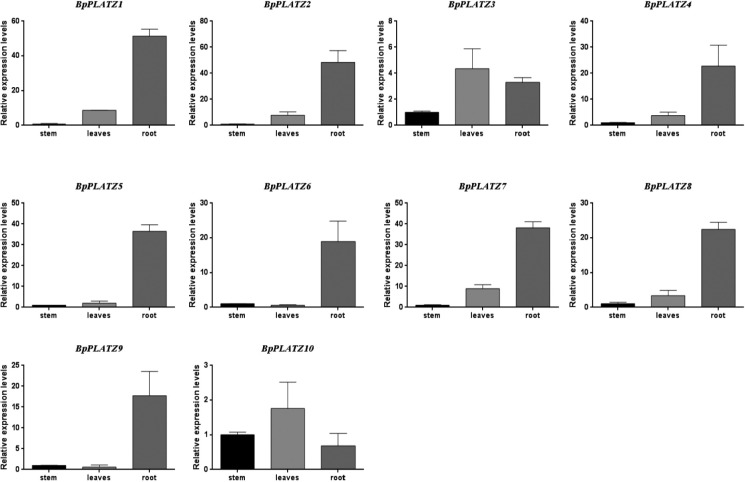
The expression of *BpPLATZs* in various parts of birch. The error lines represented the mean ± standard error of the three biological replicates of the RT-qPCR analysis.

Related studies have shown that the PLATZ transcription factor is involved in and regulates the process of plant growth and development. In this study, the RT-qPCR was used to detect the expression levels of the ten *BpPLATZ* genes in roots, stems, and leaves of birch. The results showed (Fig. 8) that the two genes, *BpPLATZ3* and *BpPLATZ10*, in group I were relatively more expressed in the stems than in the leaves and roots, while all genes in other groups were more expressed in the roots than in the leaves or stems, suggesting that they play a certain role in root development.

### RT-qPCR analysis of *BpPLATZs* under salt stress conditions

**Fig. 9.**
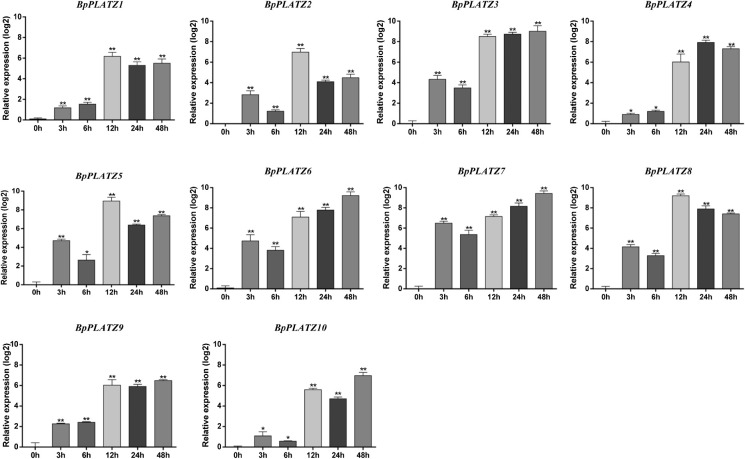
RT-qPCR analysis of *BpPLATZs* under different salt stress. The error lines represented the mean ± standard error of the three biological replicates of the RT-qPCR analysis. Significant differences between the BpPLATZ control groups (0 h) and the stress treatment groups were indicated by asterisks determined, * and ** represent significant differences from the control group, and p values are p < 0.05 (*) and p < 0.01 (**), respectively.

Under 0.2 M NaCl treatment conditions (Fig. 9), compared with the control (water treated), all of the *BpPLATZ* genes were induced expressed in the birh plantlets. Among them *BpPLATZ3*, *BpPLATZ5*, *BpPLATZ6*, *BpPLATZ7* and *BpPLATZ8* response early and their transcripts increased at 3 h after treatment and reach the peak at 12 or 48 h. *BpPLATZ1*, *BpPLATZ2*, *BpPLATZ4*, *BpPLATZ9* and *BpPLATZ10* had the late response, which were upregulated significantly at 12 h treatment. Almost all genes kept the higher expression levels after being induced. After 12 h treatment, the expression level of gene *BpPLATZ3*, *BpPLATZ6*, *BpPLATZ7* kept gradually increased, while the expression level of gene *BpPLATZ2*, *BpPLATZ5*, *BpPLATZ8* decreases, what were worth further research.

## Discussion

The *PLATZ* family genes were identified in many species, however, the functions and regulation mechanisms of the *PLATZ* family genes are not fully understood. In this study, ten members of the *PLATZ* family genes were identified in *B. platyphylla*. Compared with the identified species, the number of *PLATZ* genes in birch is similar to that of the herbaceous plant *A. thaliana* (12), the woody plant *G. biloba* (11), it accounts for about half of the gene count in *P. trichocarpa* (18), accounts for about 1/6 of the number of genes in wheat (62). However, there are large discrepancies in the genome size of these species. *B. Platyphylla* genome size (441 Mb) (Chen *et al.* 2021), is 3.53 times larger than Arabidopsis (125 Mb) (Arabidopsis Genome Initiative 2000), the genome sizes of birch and *P. trichocarpa* (480 Mb) (Tuskan *et al.* 2006), are similar, the size of the *G. biloba* genome (9.87Gb) (Liu *et al.* 2021) is 22.91 times that of *B. Platyphylla* genome, and whereas the size of the hexaploid wheat genome (13.4 to 16.23 GB) (Zhang and Qiu 2023) is 31–37 times larger than that of birch. This indicates that the number of PLATZ protein members and genome size are less relevant. Gene and genome duplications are connected to the evolution of genetic and, in turn, morphological complexity (Rensing 2014). So considering that the number of *PLATZ* family genes differences may be related to their survival or functional adaptation.

Collinearity refers to the distribution or arrangement of homologous genes within or between species, which is the most significant force driving plant evolution and a plant adaptation strategy to the external environment (Panchy *et al.* 2016). The results of collinearity analysis within species indicate that only one pair *BpPLATZ* family members have gene duplication (Fig. 2A), there was a collinear relationship between 8 birch *PLATZ* genes and 13 *P. trichocarpa PLATZ* genes, 3 birch *PLATZ* genes and 6 *A. thaliana PLATZ* genes (Fig. 2B), suggesting the genetic redundancy is low during *BpPLATZ* family evolution in birch. This also could explain the small number of *PLATZ* genes in the birch family. In most groups, gene phylogeny followed species phylogeny (Schilling *et al.* 2020). Through collinearity analysis with *A. thaliana* and *P. trichocarpa* (Fig. 2B), this suggests that *BpPLATZ1*, *BpPLATZ3* and *BpPLATZ9* may have existed before species differentiation and played an important role in the evolution of these plants. Six genes, which has no collinear gene pairs with *A. thaliana*, may be related to the difference between herbs and woody plants. These repeated gene sequence events indicate that birch and poplar, which are also woody plants, have less variation and retain more similar gene sequences during species evolution.

To understand *BpPLATZs* evolutionary relationships, a phylogenetic tree was constructed using PLATZ proteins with different species, including herbaceous plant and woody plant (Fig. 4). This study divided the *PLATZ* family genes of birch into five groups based on the grouping of *PLATZ* family genes in *P. trichocarpa*. *BpPLATZ* genes within the same group, such as *BpPLATZ1* and *BpPLATZ4* possessed similar gene structures and conserved motifs (Fig. 6), they may have similar functions during their evolutionary process. The group III only has one *BpPLATZ* gene (*BpPLATZ5*), and the predicted result of the 3D structure is different from the other nine genes (Fig. 5), so it is speculated that *BpPLATZ5* may play a special role in the development process of birch, which further confirms the accuracy of grouping. Among the five groups, only the gene number ratio of birch to *A. thaliana* in group IV, was 3:1, indicating that the *PLATZ* genes in this group may have a special function in birch, which needs to be studied. Combined with phylogenetic analysis, it was found that the same motif existed among all the BpPLATZ proteins, and the protein motif distribution was similar (Fig. 5), which confirmed that the *PLATZ* family genes of birch have homologous evolution and high conserved regions once again and conserved amino acids maintain their sequence stability under the selection of evolutionary pressure.

Zinc finger transcription factors are a relatively large family of plant transcription factors (accounting for about 15% of the total) that regulate the expression of multiple genes under abiotic stresses such as low temperature, salt, drought, osmotic stress, and oxidative stress (Kiełbowicz-Matuk 2012). Related studies have shown that the PLATZ transcription factor plays a role in salt stress response. When analyzing the *cis*-elements in the promoter of the *BpPLATZ* genes, it was also found that salt, light, drought and abolic acid activation response elements exist in all these gene promoter regions (Fig. 7), suggesting that *BpPLATZ* genes may response to salt stress, light, drought stress and specific activation of plant hormones, so as to regulate the accumulation process of secondary metabolites and environmental adaptation. Meanwhile, This study found that MYC *cis*-elements are are most rich in the ten gene promoter regions, relevant studies have shown that MYC *cis*-elements are related to plant salt tolerance, for example: the MYC element in the upstream promoter fragment of the starting codon of the *AhRabG3f* gene cloned from peanut might be a negative *cis*-element responsible for salt stress (Du *et al.* 2022). By *cis*-regulatory elements (CREs) and transcription factor binding sites (TFBSs) analyses found that LTR, MYC, [AP2; ERF], and NF-YB, which are related to salt stress, drought stress, and the response to abscisic acid (ABA) (Herwibawa *et al.* 2024). Homologous GhCLC5/16, with the highest NaCl-induced upregulation of expression and the maximum number of MYC *cis*-acting elements, might be the key members contributing to cotton Cl^–^/salt tolerance by regulating the transport, interaction and homeostasis of Cl^–^ and NO_3_^–^ (Liu *et al.* 2020). DgbHLH128 may also be involved in drought and salt stress by binding to the MYC element (Lu *et al.* 2022). So in this study, the expression of *BpPLATZs* under different durations of salt stress treatment were explored. The results showed that *BpPLATZ3*, *BpPLATZ5*, *BpPLATZ6*, *BpPLATZ7* and *BpPLATZ8* had higher expression levels than control under salt treatment (Fig. 9), which indicate that they play important roles in the regulation of salt tolerance of birch. At the same time, the expression of these genes in the root was higher than that in other tissues of birch (Fig. 8). MirMAN isolated from *Mirabilis jalapa* L. improves plant salt tolerance by promoting the development of lateral roots in *Arabidopsis* (Xu *et al.* 2023b). GmCOL1a enhances salt tolerance by promoting the accumulation of GmP5CS protein in transgenic soybean hairy roots (Xu *et al.* 2023a). LbHLH enhances salt tolerance by reducing root hair development and enhancing osmotic resistance under NaCl stress in *Limonium bicolor* (Wang *et al.* 2021). The characteristic of *BpPLATZs* high expression in roots (Fig. 8) suggests that their response to salt stress may be related to root resistance.

This study provides a data and genes basis for further understanding the evolutionary mechanism and functional traits of the *PLATZ* family genes in birch, and to lay the foundation for further study on their salt tolerant regulational mechanism. However, due to the lack of relevant experimental evidence, the specific regulatory mechanisms of these genes have not been elucidated, so the accuracy of the predicted results needs to be verified through molecular biology experiments in the later stage.

## Author Contribution Statement

CW secured funding for the study, designed the study, and revised the manuscript, YL wrote the manuscript and performed some of the assays, MY and YC analyzed all data, MZ and ZW provided the plant materials, YG, XL and CG revised the manuscript. All authors have read and approved the final version of the manuscript.

## Figures and Tables

**Table 1.** Physicochemical properties of PLATZ gene family proteins in *Betula platyphylla*

Gene name	Gene ID	Number of amino acids	Molecular weight (KD)	Theoretical PI	Total number of atoms	Instability index	Grand average of hydropathicity
BpPLATZ1	BPChr01G31955.v1.1.679	972	81.04	4.98	10333	41.39	0.743
BpPLATZ2	BPChr02G08030.v1.1.679	825	68.97	5.10	8771	42.57	0.751
BpPLATZ3	BPChr02G20753.v1.1.679	639	53.20	5.11	6658	52.11	0.879
BpPLATZ4	BPChr03G18596.v1.1.679	651	53.39	5.16	6831	41.03	0.799
BpPLATZ5	BPChr03G23087.v1.1.679	696	57.64	5.17	7441	31.24	0.728
BpPLATZ6	BPChr07G01594.v1.1.679	792	65.63	5.11	8352	42.12	0.749
BpPLATZ7	BPChr08G18129.v1.1.679	597	49.04	5.18	6304	43.88	0.853
BpPLATZ8	BPChr12G25403.v1.1.679	720	59.32	5.14	7581	36.59	0.767
BpPLATZ9	BPChr13G19857.v1.1.679	744	61.97	5.11	7835	49.28	0.776
BpPLATZ10	BPChr14G27788.v1.1.679	636	52.06	5.17	6668	37.37	0.752

**Table 2.** Secondary structure analysis and subcellular localization prediction of PLATZ gene family proteins in brich

Gene name	Alpha helix (%)	Extended strand (%)	Beta turn (%)	Random coil (%)	Subcellular localization
BpPLATZ1	21.25	12.08	1.67	65.00	cytoplasm /Nucleus
BpPLATZ2	29.93	10.58	2.19	57.30	chloroplast/Nucleus
BpPLATZ3	22.64	19.34	5.66	52.36	Nucleus
BpPLATZ4	14.35	23.61	2.31	59.72	Nucleus
BpPLATZ5	23.81	12.55	3.90	59.74	Nucleus
BpPLATZ6	25.48	14.07	4.18	56.27	Nucleus
BpPLATZ7	30.81	16.67	4.04	48.48	mitochondrion/Nucleus
BpPLATZ8	28.45	15.06	6.69	49.79	Nucleus
BpPLATZ9	24.29	10.53	2.83	62.35	Nucleus
BpPLATZ10	26.07	16.59	4.27	53.08	Nucleus
